# Ultrasound-guided fine needle aspiration cytology of Para-aortic lymph node metastasis in uterine cervical cancer: diagnostic accuracy and impact on clinical decision making

**DOI:** 10.1186/s12885-021-08492-2

**Published:** 2021-08-27

**Authors:** Junping Liu, Xin Liu, Zhengying Guo, Xiaojuan Lv, Weimin Mao, Dong Xu, Lijing Wang

**Affiliations:** 1 Department of Ultrasound, Cancer Hospital of the University of Chinese Academy of Sciences (Zhejiang Cancer Hospital), No.1 East Banshan Road, Gongshu District, Hangzhou, 310022 Zhejiang Province China; 2 Department of Pathology, Cancer Hospital of the University of Chinese Academy of Sciences (Zhejiang Cancer Hospital), No.1 East Banshan Road, Gongshu District, Hangzhou, 310022 Zhejiang Province China; 3 Department of Gynecologic Oncology, Cancer Hospital of the University of Chinese Academy of Sciences (Zhejiang Cancer Hospital), No.1 East Banshan Road, Gongshu District, Hangzhou, 310022 Zhejiang Province China; 4 Zhejiang Key Laboratory of the Diagnosis & Treatment Technology on Thoracic Oncology, No.1 East Banshan Road, Gongshu District, Hangzhou, 310022 Zhejiang Province China

**Keywords:** Cervical cancer, Para-aortic lymph node metastases, Fine needle aspiration cytology, Diagnosis accuracy, Ultrasound

## Abstract

**Objective:**

The main aim of this study was to ascertain the effectiveness of ultrasound-guided fine needle aspiration cytology (US-FNAC) in the diagnosis of para-aortic lymph node (PALN) metastasis in uterine cervical cancer and to establish its potential impact on clinical therapeutic decision making.

**Methods:**

We retrospectively reviewed clinical data from 92 patients diagnosed with cervical cancer with PALN enlargement between 2010 and 2018. Cytological results obtained with US-FNAC were classified by the same experienced cellular pathologists. Diagnostic indicators were determined on the basis of biopsy, imaging and clinical follow-up results. Univariate and multivariate analyses were used to assess the differences of influencing factors. The effect of US-FNAC on clinical decision making was evaluated.

**Results:**

Cytological results of US-FNAC were categorized as malignancy (*n* = 62; 67.4%), suspicious malignancy (*n* = 11; 12.0%), undetermined (*n* = 5; 5.4%), benign (*n* = 10; 10.9%), and inadequate (*n* = 4; 4.3%). Satisfactory biopsy samples were obtained from 95.7% of PALNs sampled (88/92). The sensitivity, specificity, positive predictive value, negative predictive value and accuracy of FNAC in distinguishing benign from malignant cases were 90.1% (95% CI: 0.809–0.953), 100% (95% CI: 0.561–1), 100% (95% CI: 0.938–1), 46.7% (95% CI: 0.223–0.726) and 90.9% (95% CI: 0.848–0.970), respectively. Univariate analysis indicated significant differences in experience of puncture physicians (radiologists) between the correct and wrong diagnosis groups (*P* < 0.05), which was further confirmed as an independent predictor of diagnostic accuracy in multivariate analysis (*p* = 0.031, OR = 0.077, 95% CI: 0.354–0.919). All patients tolerated the US-FNAC procedure well and only nine presented slight abdominal discomfort. The therapeutic strategies for 74 patients (80.4%) were influenced by US-FNAC findings.

**Conclusions:**

US-FNAC was a relatively safe and effective technique for examination of enlarged para-aortic lymph nodes and may therefore serve as a routine diagnostic tool to guide clinical decision making for management of cervical cancer.

**Supplementary Information:**

The online version contains supplementary material available at 10.1186/s12885-021-08492-2.

## Materials and methods

### Patients

**Fig. 1:**
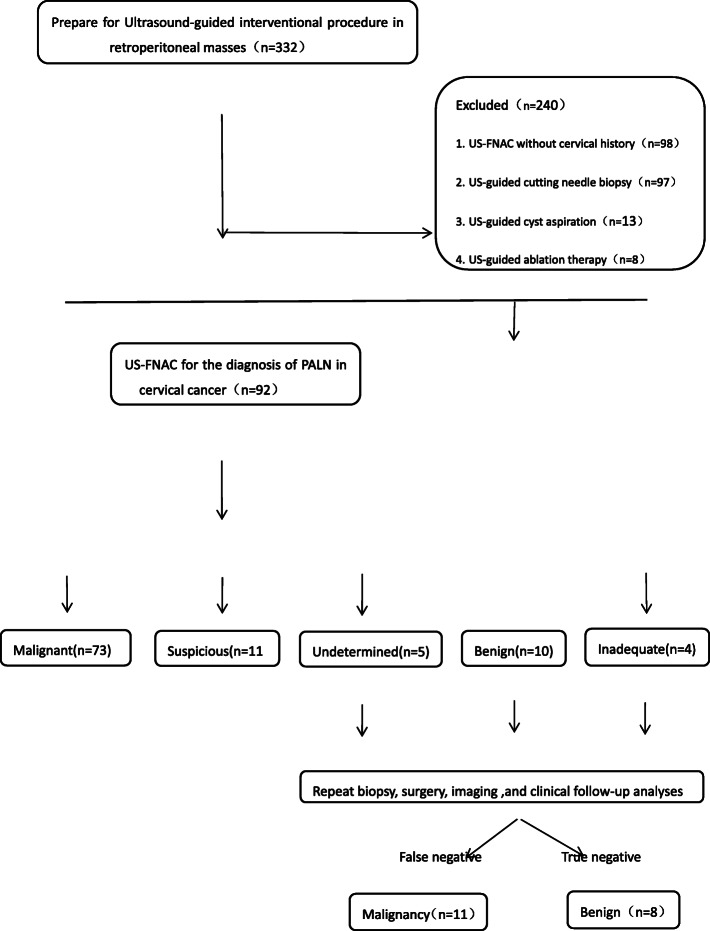
Flow chart of US-FNAC from 2010 to 2018 for diagnosis of PALN in cervical cancer

This study was approved by the Ethics Committee of Zhejiang Cancer Hospital and conducted in accordance with the ethical standards of the 1964 Helsinki Declaration. The need for informed consent from all patients was waived due to the retrospective nature of the study. We used the key words “retroperitoneal,**”** “para-aortic lymph node,” “biopsy,” and “aspiration” to screen an interventional ultrasound database between January 1, 2010, and December 30, 2018. Figure 1 depicts the workflow used for inclusion and exclusion of cases. According to the criteria used, 240 cases were excluded and we consecutively enrolled 92 patients with PALNs from cervical cancer referred to our department for US-FNAC by gynecological oncologists who required pathological results for guidance of patient treatment.

### Ultrasound examination

**Fig. 2:**
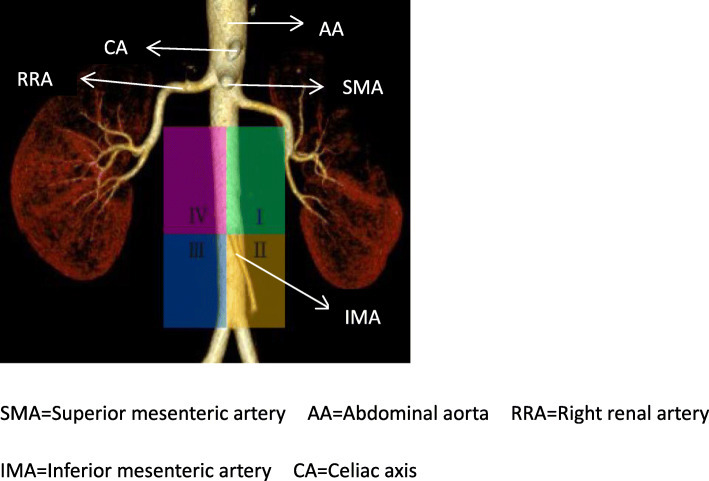
Schematic diagram of the anatomical distribution of the para-aortic lymph nodes. SMA = Superior mesenteric artery AA = Abdominal aorta RRA = Right renal artery. IMA = Inferior mesenteric artery CA = Celiac axis

Initially, each patient underwent a detailed ultrasound examination to determine the feasibility of FNAC. The size, number, location and characteristics of the affected lymph nodes were assessed using GE-E9 (5–16 MHz) and Esaote MyLab Twice (4–12 MHz). The locations of PALNs for puncturing were divided into four regions (designated levels I–IV) based on anatomical structure [2] (Fig. 2). The nodule size was measured in the central section of the cross-sectional image, taking the diameter perpendicular to the abdominal aorta as the length. Lymph nodes with safe puncture paths and highly suspicious morphology were considered suitable puncture targets. During US-FNAC, necrotic areas were avoided as much as possible. In order to avoid damaging vessels, color Doppler was used to confirm the safest puncture path.

### US-FNAC procedure

**Fig. 3:**
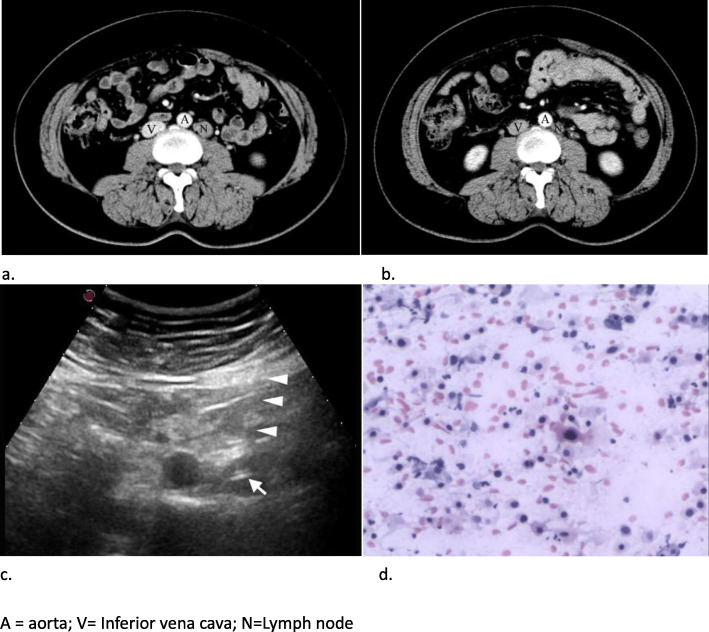
US-FNAC and therapeutical effect of a para-aortic lymph node. **a** a 49-year-old woman with metastatic squamous carcinoma from cervical cancer presents with a 16 mm Lymph node located left paraaortic region above the inferior mesenteric artery (I). **b** After 5 months of radiotherapy, the size of lymph nodes are significantly reduced. **c** Needle tip (arrow) is clearly defined as being within the Lymph node and conventional US displays the pass of the puncture needle through the mesentery or bowels (arrowhead). **d** The FNAC results was diagnosed as metastatic squamous cell carcinoma with scattered atypical cells, necrosis and cytokeratinization (HE stain,× 20). = aorta; V = Inferior vena cava; N = Lymph node

Prior to puncture, all patients were subjected to coagulation status screening (international standardized ratio was < 1.6, prothrombin time < 15 s, partial thromboplastin time < 45 s, platelet count > 80,000 per mm^3^). Informed consent for the US-FNAC procedure was obtained from all patients. Punctures were carried out by five radiologists familiar with the FNAC process. The site of puncture was marked on the skin and the area cleaned with povidone-iodine solution. Following local anesthesia with 2% lidocaine, US-FNAC was performed using a 20–23 gauge needle (Hakko Co, Japan) and attached to an empty 5 mL plastic syringe using negative suction with at least one pass (Fig. 3). All operators used a free-hand technique (without biopsy attachment). On-site cytology evaluation was not performed in our department. Patients were required to be in the hospital and monitored for vital signs and complications.

Cellular material was subsequently expelled onto a glass slide that was immediately immersed in 95% alcohol. In some cases, cell blocking was conducted. Specimens were sent to the cytology department and stained using the hematoxylin and eosin method to determine color changes in the refractive index of the fine structure of the cell. Final cytology diagnosis was made by the same cellular pathologists with more than 10 years of experience.

### FNAC categorization and final diagnosis

FNAC results were classified into the following groups: unsatisfactory/non-diagnostic material for diagnosis (i, inadequate group), benign or reactive change (ii, benign group), atypical cells present but indeterminate for benign and malignancy (iii, undetermined group), suspicious for malignancy (iv, suspicious group), and positive for malignancy (v, malignant group). An unsatisfactory/non-diagnostic result (i) was defined in cases where the sample consisted only of the blood component or an acellular specimen. Results were considered benign (ii) when mature lymphocytes in terms of morphology or linear colloid-like substance (foreign body granuloma) were obtained from aspirated nodules. Undetermined results (iii) were obtained for samples that consisted exclusively of a small amount of atypical cell components but no conclusive benign and malignancy status. In cases where cytological findings were indicative of suspected or unequivocal malignancy, diagnosis was “suspicious for malignancy” (iv) and “positive for malignancy” (v), respectively. Unsatisfactory/non-diagnostic results (i) were defined as “inadequate” “and the remaining results as “adequate/satisfactory” specimens. Final diagnoses were confirmed in cases of i, ii and iii via repeat biopsy, surgery, imaging, clinical indicators, and clinical follow-up analyses [15].

### Statistical analysis

Cases of malignancy (iv, v) were considered true-positive diagnoses. In cases where shrinkage, disappearance, or stationary condition was presented for a period of 12–24 months without specific treatment during follow-up imaging or if subsequent re-biopsy or surgery indicated a benign pathologic result (i, ii, iii), a true-negative result was recorded. A false-negative result (i, ii, iii) was defined in cases where nodes were obviously enlarged in follow-up imaging, malignant results were confirmed with surgery and re-biopsy or clinical data were highly suggestive of malignancy. False-negative results were considered incorrect diagnoses. The evaluation index of diagnostic testing incorporated specificity, sensitivity, negative predictive value (NPV), positive predictive value (PPV) and accuracy parameters, which were calculated based on previous definitions.

Continuous variables were expressed as means±standard deviation and statistical analysis performed using ANOVA. Categorical variables were presented on the basis of frequency and percentage and the analysis performed using chi-square or Fisher’s exact test. Univariate analysis (chi-square or a Fisher exact test) was conducted to investigate the relationships among experience, age, body mass index, nodal size, nodal number, lesion location, needle size, cell block, nature and period of treatment. Multivariate logistic regression analysis was additionally used to determine variables independently contributing to the diagnostic yield of sampling. Differences were considered statistically significant at *P* < 0.05. SPSS software (version 21.0 for Windows; SPSS, Chicago, IL, USA) was used for all statistical analyses.

## Results

### General clinical data

Overall, 24 patients with cervical cancer were excluded (Fig. 1). In 16 of the 24 cases, enlarged lymph nodes observed via CT imaging could not be visualized with conventional ultrasound. In five of the cases, US-FNAC could not be performed due to small node sizes (< 10 mm). There were no safe access paths for puncture to avoid injury to major vessels in three patients. Among the 92 included cases, patient ages ranged from 25 to 80 years (mean: 51.5 ± 10.8 years). The node sizes ranged from 11 mm to 28 mm (average: 17.1 ± 4.0 mm). Body mass index (BMI) ranged from 14.6 to 32.0 (mean: 22.0 ± 3.4). In 79 cases (85.9%), multiple nodes were present while a solitary node was detected in 13 cases (14.1%). The 20G and 21G fine needles were used for the US-FNAC procedure in 60 cases. Biopsies was performed with 22G and 23G needles using a suction syringe in 32 cases.

In terms of anatomical grouping, PALNs were divided into left and right distributions based on the abdominal aorta. The inferior mesenteric artery was taken as the boundary level dividing the upper and lower regions (Fig. 2). Overall, 55 cases were in the left para-aortic region (level I: 27 cases and level II: 28 cases) and 37 cases in the right para-aortic region (level III: 29 cases and level IV: 8 cases; Table 1).

**Table 1:** Comparision of demographics and lesion distribution among the five groups

Characteristics Group	Total (*n* = 92)	Malignant (*n* = 62)	Suspicious (*n* = 11)	Undetermined (*n* = 5)	Benign (*n* = 10)	Inadequate (*n* = 4)	*P* value
Age[$\overline{\mathrm{x}}$ ±s](year)	51.5 ± 10.8	50.9 ± 9.4	58.6 ± 15.5	52.8 ± 15.5	46.0 ± 8.8	54.5 ± 7.4	0.087
Body mass index	22.0 ± 3.4	21.7 ± 3.4	23.7 ± 5.0	22.9 ± 1.6	21.5 ± 2.1	20.8 ± 1.6	0.302
Lesion size (mm)	17.1 ± 4.0	17.6 ± 3.8	18.0 ± 4.7	16.0 ± 4.0	14.6 ± 3.1	13.8 ± 3.2	0.061
Nodal number							1.000
solitary	13 (14.1%)	7 (7.6%)	1 (1.1%)	0 (0.0%)	4 (4.3%)	1 (1.1%)	
multiple	79 (85.9%)	55 (59.8%)	10 (10.9%)	5 (5.4%)	6 (6.5%)	3 (3.3%)	
Location							
Left Paraaortic							
Above the IMA(I)	27 (29.3%)	18 (19.5%)	5 (5.4%)	1 (1.1%)	0 (0.0%)	3 (3.3%)	0.670
Below the IMA (II)	28 (30.5%)	19 (20.7%)	4 (4.3%)	1 (1.1%)	3 (3.3%)	1 (1.1%)	
Right Paraaortic							
Below the IMA (III)	29 (31.5%)	18 (19.5%)	2 (2.2%)	3 (3.3%)	6 (6.5%)	0 (0.0%)	1.000
Above the IMA (IV)	8 (8.7%)	7 (7.6%)	0 (0.0%)	0 (0.0%)	1 (1.1%)	0 (0.0%)	
Needle gauge							
≤ 21G(20G,21G)	60 (65.2%)	40 (43.5%)	8 (8.7%)	4 (4.3%)	6 (6.5%)	2 (2.2%)	1.000
≥ 22G(22G,23G)	32 (34.8%)	22 (23.9%)	3 (3.3%)	1 (1.1%)	4 (4.3%)	2 (2.2%)	
Cell block							0.483
Yes	31 (33.7%)	15 (16.1%)	9 (9.9%)	1 (1.1%)	3 (3.3%)	3 (3.3%)	
No	61 (66.3%)	47 (51.1%)	2 (2.2%)	4 (4.3%)	7 (7.6%)	1 (1.1%)	
Final diagnosis							0.110
Malignant	84 (91.3%)	62 (67.4%)	11 (11.9%)	5 (5.4%)	3 (3.3%)	3 (3.3%)	
benign	8 (8.7%)	0 (0.0%)	0 (0.0%)	0 (0.0%)	7 (7.6%)	1 (1.1%)	
Experience (years)	5.5 ± 3.1	6.2 ± 3.0	4.4 ± 2.9	2.4 ± 2.1	5.3 ± 3.7	2.8 ± 2.2	0.011

Experience refers to the years of engaging in interventional ultrasound

### Cytological findings and final diagnosis

Biopsy-based cytological findings were as follows: malignant (*n* = 62; 67.4%), suspicious (*n* = 11; 12.0%), undetermined (*n* = 5; 5.4%), benign (*n* = 10; 10.9%), and unsatisfactory (*n* = 4; 4.3%). In two undetermined and one inadequate cases, repeat US-FNAC was performed, which provided sufficient evidence for confirmed diagnosis of squamous cell carcinoma. The remaining three undetermined and two inadequate cases were confirmed as malignant based on clinical follow-up and imaging analyses. However, a final benign diagnosis was established in one inadequate case due to obvious tumor shrinkage at 4 months follow-up. Therefore, among nine samples, eight were finally diagnosed as malignant (false-negative) and 1 as benign (true-negative) (Table 2).

**Table 2:** The diagnostic categorizations of initial US-FNAC in PLANs and final diagnosis

Diagnosis category Cytology by US-FNAC	Final diagnosis of PALNs, n		
	Malignant (***n*** = 84)	Nonmalignant (***n*** = 8)	Total (***n*** = 92)
Malignant	62 (67.4%)	0 (0.0%)	62
Suspicious	11 (11.9%)	0 (0.0%)	11
Undetermined	5 (5.4%)	0 (0.0%)	5
Benign	3 (3.3%)	7 (7.6%)	10
inadequate	3 (3.3%)	1 (1.1%)	4

Final diagnoses were confirmed by at least two experts, including radio-oncologists, imaging specialists, and pathologists [15]. *US-FNAC* ultrasound guided fine-needle aspiration cytology, *PALNs* para-aortic lymph nodes

**Fig. 4:**
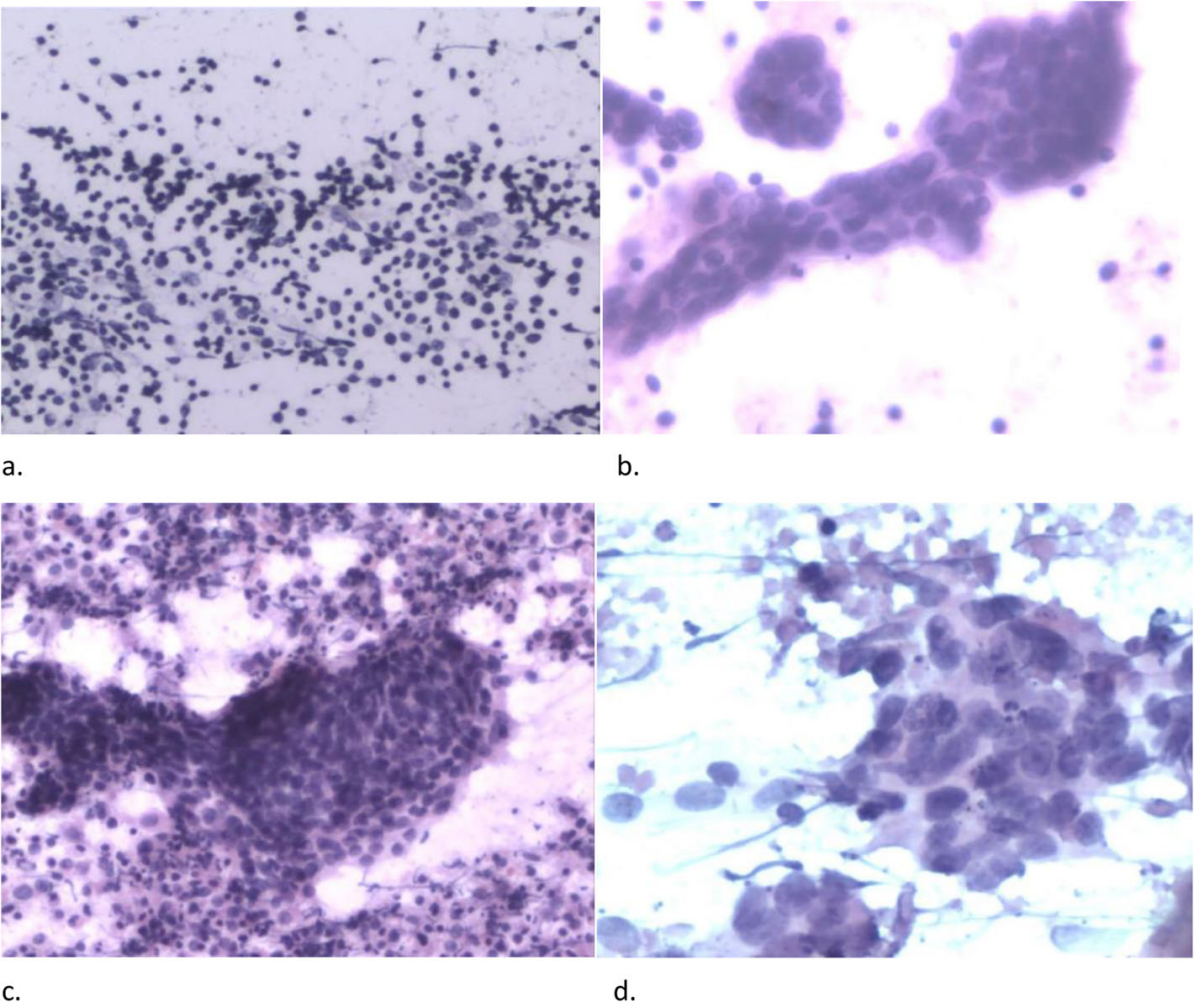
The cytological morphology diagnosis of US-FNAC in para-aortic lymph nodes. **a** Cytology smear showing scattered lymphocytes and histiocytes with mixed morphology is reported as benign results (HE stain,× 10). **b** Cytology smear showing glandular clusters with atypical epithelial cells scattered in the lymphocyte background is diagnosed as metastatic adenocarcinoma (HE stain,× 20). **c** In the degenerated necrotic lymphoid tissue, there are clusters of atypical epithelial cells arranged in whirlpool, showing keratinization tendency, which is regarded as metastatic squamous cell carcinoma (HE stain,× 20). **d** In a large necrotic background, poorly differentiated cancer cells were seen in clusters, with obvious nucleoli and unclear differentiation tendency, which is considered as metastatic undifferentiated carcinoma (HE stain,× 40)

Ten cases were diagnosed as benign based on US-FNAC (Fig. 4a). Three of the benign cases were further subjected to surgical resection for therapeutic and diagnostic purposes, leading to definitive malignant diagnosis. The final diagnosis of the remaining seven benign FNAC cases was confirmed as benign based on clinical follow-up and imaging. No false-positive results were obtained. Therefore, final diagnosis of the 10 benign samples re-examined was malignant for three (false-negative) and benign for seven (true-negative) cases.

Eighty-eight (95.7%) biopsy specimens were regarded as satisfactory samples by more than 10 experienced cytopathologists. Among the 73 malignant specimens diagnosed using FNAC, 62 were unambiguously confirmed as malignant, including five adenocarcinoma (Fig. 4b), 39 squamous cell carcinoma (Fig. 4c), and 18 undifferentiated metastatic carcinoma (Fig. 4d), while 11 were diagnosed as probable metastatic carcinoma. According to the definition (iv, v), all 73 cases were finally diagnosed as malignant (true-positive).

### Accuracy of US-FNAC

Due to variable definitions of false-negative and true-negative adopted previously for assessing diagnostic accuracy [16], we used the analytical methods described below for data presentation in this study. Upon exclusion of the four inadequate cases from our analyses and taking undetermined specimens as negative cytological results, the true-positive, false-positive, false-negative and true-negative values obtained were 73, 0, 8 and 7, respectively. The sensitivity, specificity, PPV, NPV and accuracy of FNAC in distinguishing benign from malignant cases were determined as 90.1% (95% CI: 0.809–0.953), 100% (95% CI: 0.561–1), 100% (95% CI: 0.938–1), 46.7% (95% CI:0.223–0.726) and 90.9% (95% CI:0.848–0.970), respectively. Upon exclusion of the five cases with undetermined cytologic diagnosis, true-positive, false-positive, false-negative and true-negative values obtained were 73, 0, 3 and 7, respectively. The sensitivity, specificity, PPV, NPV and accuracy of FNAC in distinguishing benign from malignant cases were determined as 96.1% (95% CI:0.881–0.990), 100% (95% CI:0.561–1), 100% (95% CI: 0.938–1), 70.0% (95% CI: 0.354–0.919) and 96.4% (95% CI: 0.923–1), respectively. We observed no significant differences between the two methods (Table 3).

**Table 3:** The diagnostic performance of US-FNAC for PLANs in cervical cancer

Diagnostic index	Inclusion of Undetermined	Exclusion of Undetermined	*P* value
Sensitivity, %(n)	90.1%(73/81)	96.1%(73/76)	0.146
Specificity, %(n)	100%(7/7)	100%(7/7)	1.000
PPV, %(n)	100%(73/73)	100%(73/73)	1.000
NPV, %(n)	46.7%(7/15)	70.0%(7/10)	0.250
Accuracy, %(n)	90.9%(80/88)	96.4%(80/83)	0.145

*PPV* Positive predictive value, *NPV* Negative predictive value

### Factors influencing diagnostic accuracy

Correct diagnosis (*n* = 81) and incorrect diagnosis (false negative cases, *n* = 11) groups in relation to variables affecting diagnostic accuracy are presented in Table 4. Experience refers to the years of engaging in interventional ultrasound. Univariate analysis revealed that the diagnostic accuracy of US-FNAC was significantly related to experience (5 y vs. > 5 y; *p* = 0.008), but not age, body mass index (≤24 vs. > 24), nodal size (≤15 mm vs. > 15 mm), nodal number (multiple vs. solitary), lesion location (left para-aortic vs. right para-aortic), needle size (≤21G vs. ≥22G), cell block (yes vs. no), nature (malignant vs. benign) and period treatment(2010–2015 vs. 2016–2018) (*p* > 0.05). Multivariate logistic regression analysis further validated the correlation between more experienced operators and correct diagnosis (*p* = 0.031, OR = 0.077, 95% CI: 0.354–0.919; Table 4).

**Table 4:** Variate analysis of the factors influencing diagnostic yield

Variables	Correct diagnosis (*n* = 81)	Wrong Diagnosis (*n* = 11)	Univariate analysis	Multivariate analysis		
			*P* value	OR	95%CI	*P* value
Age[$\overline{\mathrm{x}}$ ±s (range)](year)	51.3 ± 10.8	53.1 ± 11.0	0.614	1.039	0.96–1.124	0.344
Nodal size, mm			0.322	0.333	0.073–1.510	0.154
> 15 mm	52	5				
≤ 15 mm	29	6				
Nodal number			0.353	–	–	–
multiple	68	11				
solitary	13	0				
Nodal location				0.553	0.106–2.877	0.481
Right Paraaortic	49	5	0.352			
Left Paraaortic	32	6				
Needle gauge			0.182	1.090	0.235–5.049	0.913
≤ 21G	26	6				
≥ 22G	55	5				
Body mass index (BMI)			0.446	0.187	0.015–2.392	0.197
≤ 24	61	10				
> 24	20	1				
Cell block			0.498	1.470	0.212–10.20	0.697
Yes	26	5				
No	55	6				
Nature of lymph node			0.589	–	–	–
Malignant	73	11				
Benign	8	0				
Experience (years)			0.008	0.077	0.007–0.793	0.031
≤ 5	37	10				
> 5	44	1				
Period			0.197	2.501	0.372–16.83	0.346
2010–2015	43	3				
2016–2018	38	8				

### Complications

All patients tolerated the US-FNAC procedure well. No major complications were encountered during the procedure, such as bleeding, perforation and infection. Nine patients presented with slight abdominal discomfort, which was relieved after 1 hour of clinical observation. No further clinical treatment was required.

### Impact of US-FNAC on clinical decision making

Radiotherapy, chemotherapy, and surgery (laparoscopy or laparotomy) are commonly used for patients with PALN metastases from cervical cancer. After US-FNAC, among 73 patients with cytological malignancy, 59 were subjected to PALN radiotherapy and systemic chemotherapy and only nine received chemotherapy. PALN sizes were significantly reduced after treatment. Surgery was performed in five patients. Among the 10 patients with benign cytological diagnosis, three cases with features highly suggestive of malignancy based on PET/CT imaging underwent surgical biopsy and were re-diagnosed as metastatic squamous cell carcinoma. The remaining seven patients required only observation. After 12–24 months of follow-up, three patients displayed reduction in PALN size, while sizes remained stable in four patients. Among the four unsatisfactory samples and five undetermined cases, five were treated with PALN radiotherapy and systemic chemotherapy on the basis of PET/CT imaging data and three received the same therapy according to secondary US-FNAC results, which led to significant reduction in PALN size. One case with no indication of malignancy in PET/CT imaging was selected for clinical follow-up, resulting in a decrease in PALN size after 4 months. Overall, application of the US-FNAC tool affected the therapeutic methods used in 74 (80.4%) cases.

## Discussion

Results from the current study indicated that US-FNAC was useful as sonographic guidance for detection of PALNs in cervical cancer with diagnostic accuracy of 90.9%, which was superior to the accuracy rate of 86.1% reported previously by Shao et al. [11] from analysis of FNAC results via CT-guided percutaneous needle biopsy of 315 patients with retroperitoneal lymphadenopathy. However, endoscopic US-guided FNAC for preoperative PALN staging in patients with pancreatobiliary cancer by Yeow et al. [17] showed that EUS-FNA had higher accuracy (98.6%) for diagnosis of PALN metastasis, which could have clinical benefits in terms of precluding unnecessary surgery. In the present study, diagnostic sensitivity of 90.1% and accuracy of 90.9% of US-FNAC were comparable with previous findings of Stattaus et al. [18] (95.2 and 95.9% for undetermined retroperitoneal masses) and Avritscher et al. [19] (91.4 and 92.8% for pulmonary hilar lymph nodes).

One advantage of ultrasound guidance is that the needle tip is displayed in real time throughout the procedure and always remains within the lesion during sampling, thus minimizing contamination with blood and extraneous tissue (movie 1). Another advantage is that the manipulator can regulate almost any anatomical plane by angling and rotating the transducer to avoid visible blood vessels and important organs. When the needle tip is no longer visible with US during the procedure, gentle shaking of the puncture needle can facilitate tracking of the tip position (movie 2). However, US-FNAC was not performed in 16 of the 24 excluded cases in our study as the enlarged lymph nodes observed in CT imaging could not be visualized with conventional ultrasound, possibly due to small nodules, intestinal gas interference and flake growth of PALNs.

All patients underwent the US-FNAC procedure with no apparent complications. Perforation, a worrying complication often experienced during the process of abdominal FNAC, did not occur, and therefore no antibiotics were required following biopsy, consistent with earlier studies [12–14]. Owing to the considerable difficulty in distinguishing bowel from mesentery or omentum after transducer compression, we did not make an extensive effort to monitor the presence of bowels in the puncture path. Overall, the US-FNAC procedure appeared safe for clinical application.

Similar to EUS-FNA [17], percutaneous US-FNAC for PALNs is a highly challenging technique that is difficult to master for achievement of acceptable diagnostic accuracy. The success rate of US-FNAC is directly associated with the radiologist’s experience in terms of years of engaging in interventional ultrasound. In the current study, among the 11 cases of incorrect diagnosis, nine of the operators had less than 3 years of experience. Practitioners must undergo a lengthy learning curve to acquire the appropriate skills to obtain good views of the target node, including selection of a suitable size needle and proper knowledge of the FNAC operating technique. Accordingly, we recommend that complex techniques should only be performed by experienced individuals with professional biopsy course training. Subsequent research [by our group] will focus on the learning curve pathway of FNAC as a reference for training.

In this study, the US-FNAC approach influenced clinical decision making for 74 patients (80.4%). If patients exhibited PALN enlargement, para-aortic irradiation should be provided only in cases of pathological evidence of PALN metastasis to avoid spinal cord inhibition and intestinal toxicity. Encouragingly, our US-FNAC results were not false-positive, consistent with the findings of Fisher et al. [13]. If patients with cytologically benign PALN are considered for conservative observation, clinical and imaging data should be integrated [15]. Three patients with initial benign cytological diagnosis were subsequently diagnosed as metastatic squamous cell carcinoma based on surgical biopsy in our experiments. One possible interpretation for this finding is that the needle passes may not have sampled metastases owing to microscopic cancer focus. Another possibility is sampling error, whereby surgically excised metastatic PALNs were not the same aspirated lymph node. In cases where inadequate specimens were obtained, oncologists should combine clinical data and re-apply US-FNAC. Although treatment decisions are based on PET/CT imaging [15] in some patients, pathological evidence should be obtained before chemoradiotherapy to reduce unnecessary medical disputes over treatment.

The strengths of this study included the relatively large number of cervical cancer patients with PALN enlargement, concerning a high proportion with small PLANs in size. To our knowledge, this study is the first to evaluate the specific factors influencing diagnostic yield in large samples and highlight an independent predictor of diagnostic accuracy. Moreover, we have established the contributory role of US-FNAC in clinical decision making for the first time.

Our investigation also had a number of limitations that need to be considered. First, the study was retrospective in nature and had selectivity bias. Second, diagnosis and treatment of a number of patients were based on clinical follow-up and imaging data. Furthermore, this was a single-center study and the technical level was different. Ultimately, overall survival analysis was beyond the scope of this study and will be the focus of subsequent research.

In conclusion, US-FNAC was a safe and convenient technique that was widely available. The diagnostic accuracy of US-FNAC was high and the main influencing factor was the experience of the operator. Therefore, it is strongly recommended that operators undergo formal standardized training and certification of qualification. Furthermore, no false-positives and low-false negatives were obtained using this method, supporting its utility in clinical decision-making processes. The US-FNAC technique should therefore be added to standard pretherapy workup for cases of cervical cancer with enlarged para-aortic lymph nodes.

## Supplementary Information

**Additional file 1: Movie 1.** Manifested that the needle tip was displayed in real time throughout the procedure and the needle tip always remains within the lesion during sampling.

**Additional file 2: Movie 2.** Showed that when the needle tip was not clearly visualized, shaking the puncture needle was beneficial to the display of the needle tip.

## Data Availability

The datasets used and/or analyzed during the current study are not publicly available due privacy rules but are available from the first author on reasonable request at the following email address liujp85@zjcc.org.cn

## Abbreviations

US-FNAC: Ultrasound guided fine-needle aspiration cytology
PALN: Para-aortic lymph node DW-MRI Diffusion-weighted MR imaging
PET/CT: Positron emission tomography and computed tomography
IMA: Inferior mesenteric artery
